# Feasibility of Gynaecologist Led Lynch Syndrome Testing in Women with Endometrial Cancer

**DOI:** 10.3390/jcm9061842

**Published:** 2020-06-12

**Authors:** Neil A. J. Ryan, Louise Donnelly, Katie Stocking, D. Gareth Evans, Emma J. Crosbie

**Affiliations:** 1 Division of Evolution and Genomic Medicine, University of Manchester, St Mary’s Hospital, Manchester M13 9WL, UK; neilryan@nhs.net (N.A.J.R.); gareth.evans@mft.nhs.uk (D.G.E.); 2 Division of Cancer Sciences, Faculty of Biology, Medicine and Health, University of Manchester, St Mary’s Hospital, Manchester M13 9WL, UK; 3 Nightingale and Prevent Breast Cancer Research Unit, Manchester University NHS Foundation Trust, Manchester M23 9LT, UK; louise.gorman@manchester.ac.uk; 4 NIHR Greater Manchester Patient Safety Translational Research Centre, University of Manchester, Manchester M13 9PL, UK; 5 Centre for Biostatistics, Faculty of Biology, Medicine and Health, University of Manchester, St Mary’s Hospital, Manchester M13 9WL, UK; katie.stocking@manchester.ac.uk; 6 Manchester Centre for Genomic Medicine, Manchester University NHS Foundation Trust, Manchester Academic Health Science Centre, Manchester M13 9WL, UK; 7 Division of Gynaecology, Manchester University NHS Foundation Trust, Manchester Academic Health Science Centre, Manchester M13 9WL, UK

**Keywords:** Lynch syndrome, consent, anxiety, germline testing, endometrial cancer

## Abstract

A barrier to Lynch syndrome testing is the need for prior genetic counselling, a resource demanding process for both patients and healthcare services. We explored the impact of gynaecologist led Lynch syndrome testing in women with endometrial cancer. Women were approached before surgery, on the day of surgery or during routine follow up. Lynch syndrome testing was offered irrespective of age, family history or tumour characteristics. Women’s reasons for being tested were explored using the Motivations and Concerns for GeNEtic Testing (MACGNET) instrument. The short form State-Trait Anxiety Inventory (STAI-6) was used to measure anxiety levels. Only 3/305 women declined Lynch syndrome testing. In total, 175/220 completed MACGNET and STAI-6 psychological instruments. The consent process took an average of 7 min 36 s (SD 5 min 16 s) to complete. The point of care at which consent was taken (before, day of surgery, during follow up) did not influence motivation for Lynch syndrome testing. Anxiety levels were significantly lower when women were consented during follow up (mean reversed STAI-6 score 32 vs. 42, *p* = 0.001). Anxiety levels were not affected by familial cancer history (*p* = 0.41). Gynaecologist led Lynch syndrome testing is feasible and may even be desirable in endometrial cancer, especially when offered during routine follow up.

## 1. Introduction

Endometrial cancer is the most common gynaecological cancer in developed countries and its incidence is rising [1]. Most cases are linked to lifestyle and reproductive factors but a significant minority is caused by Lynch syndrome. Lynch syndrome is an autosomal dominant cancer predisposition syndrome arising from a dysfunctional mismatch repair (MMR) system [2]. Inherited pathogenic variants in MMR genes *MLH1*, *MSH2 (or EPCAM)*, *MSH6* or *PMS2* predispose carriers to multiple malignancies, particularly endometrial, colorectal and ovarian cancers, which typically occur at younger ages than sporadic tumours of the same sites [3]. Endometrial cancer is often the first manifestation of Lynch syndrome in women, and as such offers an important diagnostic opportunity [4]. Lynch syndrome-associated endometrial tumours are characterized by heavy immune cell infiltrates and thus are exquisitely sensitive to immunotherapy [5]. Further, a Lynch syndrome diagnosis enables participation in colorectal surveillance programmes and aspirin chemoprevention, strategies proven to reduce the risk of dying from subsequent cancers [3,6]. The benefits of diagnosis extend to close family members, who can access Lynch syndrome testing themselves and engage in risk reducing reproductive choices and preventive gynaecological surgery [7].

The potential for these interventions to save lives supports routine Lynch syndrome testing for all endometrial cancer patients, especially because selecting women by age, family history or tumour characteristics misses cases of Lynch syndrome [8]. The routine use of sequential tumour-based tests to triage women for definitive germline testing has been proposed as the most effective and cost-effective method of identifying the 3% of women with Lynch syndrome-associated endometrial cancer [9,10]. Initial tumour-based tests, routinely performed to diagnose, stage and inform individualized treatment plans, do not require explicit patient consent. However, it is widely held that women undergoing definitive germline testing should first undergo professional genetic counselling to understand the implications of testing positive [11]. This requirement is a significant barrier to wide scale implementation of routine testing, placing a considerable burden on already overstretched genetic services, yet the majority of women are expected to test negative for Lynch syndrome. Alternative strategies, including gynaecologist led testing and the referral to genetic services only those women who test positive for a germline pathogenic variant, have not been studied in Lynch syndrome-associated endometrial cancer before. The optimal timing for seeking informed consent and its impact on uptake, anxiety levels and motivation for testing is also not known.

The aim of this study was to assess the feasibility of gynaecologist led Lynch syndrome testing in women with endometrial cancer. We measured uptake, motivations and anxiety levels associated with being asked about Lynch syndrome testing as part of women’s routine endometrial cancer care.

## 2. Materials and Methods

### 2.1. Participants

Consecutive women with suspected or known endometrial cancer undergoing treatment at a large specialist gynaecological cancer service in the North West of England were invited to participate in the Proportion of Endometrial Tumours Associated with Lynch Syndrome (PETALS) study. This study, described in detail elsewhere [12], offered Lynch syndrome testing to unselected endometrial cancer patients, irrespective of their age, family history or tumour characteristics. All tumours underwent microsatellite instability (MSI) testing, immunohistochemistry (IHC) for MMR status and when MLH1/PMS2 IHC loss was present, targeted *MLH1* methylation testing. Women provided a blood sample at enrolment, however, germline testing was only carried out if tumour triage was positive (MSI-high or MMR-deficient with normal *MLH1* methylation) and/or women were aged ≤50 years or had a strong personal /family history of Lynch syndrome-associated tumours. The PETALS study was sponsored by the University of Manchester and approved by the North West Research Ethics Committee (ref 15/NW/0733). The study was prospectively registered on the Cancer Research UK clinical trial database (ref 13595).

Women were approached in outpatient clinics before surgery, on the day of surgery or during routine follow up for endometrial cancer. Written, informed consent for Lynch syndrome testing was obtained by a gynaecological oncology consultant or senior trainee involved in the patient’s clinical care. Both underwent bespoke, individualised training with a registered genetic counsellor and clinical geneticist to learn how to consent women for Lynch syndrome testing. This involved a targeted one-hour training session and opportunities to observe their clinical practice over two cancer genetic clinics. Participants were given written information about Lynch syndrome and the implications of testing positive were explained, including the increased lifetime risk of cancer, recommendations for colorectal surveillance, aspirin chemoprevention and cascade testing of family members. Women were given an appointment with a registered genetic counsellor if they tested positive for a Lynch syndrome pathogenic variant.

The time taken to consent for Lynch syndrome testing was recorded. The time started when Lynch syndrome testing was introduced and stopped when the participant signed the consent form. Whether consent was taken before surgery, on the day of surgery or during routine follow up was recorded. ‘Day of surgery’ included the period between the date of surgery and the fifth post-operative day. The number of participants who accepted or declined Lynch syndrome testing was recorded.

### 2.2. Familial and Psychological Assessments

Detailed clinical and family histories were taken from every participant. A positive family history was defined as one first degree relative, or two second degree relatives with colorectal or endometrial cancer. Participants were invited to complete an optional validated questionnaire to measure their motivations and general anxiety state at the time of consenting to Lynch syndrome testing. Motivations were recorded using the Motivations And Concerns for GeNEtic Testing (MACGNET) instrument [13]. This has five subscales: (1) Gaining knowledge for cancer prevention and obtaining information to guide medical management (B1–B11); (2) Evaluation of partner’s influence on undergoing genetic testing (B12–B15); (3) Planning for the future (B16–B18); (4) Ability to cope with testing results (B19–B24); (5) Fear of discrimination. The fifth subscale, which refers to the impact on healthcare insurance, was excluded as women were recruited from the National Health Service (NHS) in the UK. The six item short form State-Trait Anxiety Inventory (STAI-6) was used to measure state anxiety at the time of consent [14]. The STAI-6 lists six emotional states and asks participants to indicate their degree of alignment on a four-point scale.

### 2.3. Statistical Analysis

Descriptive statistics for continuous data were prepared using means and standard deviations for parametric and medians with interquartile ranges for non-parametric data. For categorical data, proportions and frequencies were given. Formal comparisons were made using a One-Way ANOVA or Kruskal–Wallis test for continuous data, and Chi-Squared or Fisher’s Exact Test for categorical variables. For psychological variables, scores of 0 were treated as missing data. Participants with <50% missing data had their total subsection score imputed using mean substitution [15]. Where >50% data were missing, questionnaires were considered incomplete and disregarded. Comparisons were made between the patient groups using a One-Way ANOVA or Kruskal–Wallis test. A post-hoc pairwise comparison was carried out using a Bonferroni adjustment where appropriate. The impact of family history on the degree of anxiety was analysed using a Mann–Whitney U Test. In all analyses, a *p*-value of <0.05 was considered statistically significant. Statistical analyses were carried out using Stata 14 (StataCorp. 2015. *Stata Statistical Software: Release 14*. StataCorp LP, College Station, TX, USA) and GraphPad Prism version 7.0a for Mac OS (GraphPad Software, La Jolla, CA, USA).

## 3. Results

### 3.1. Study Participants

**Figure 1:**
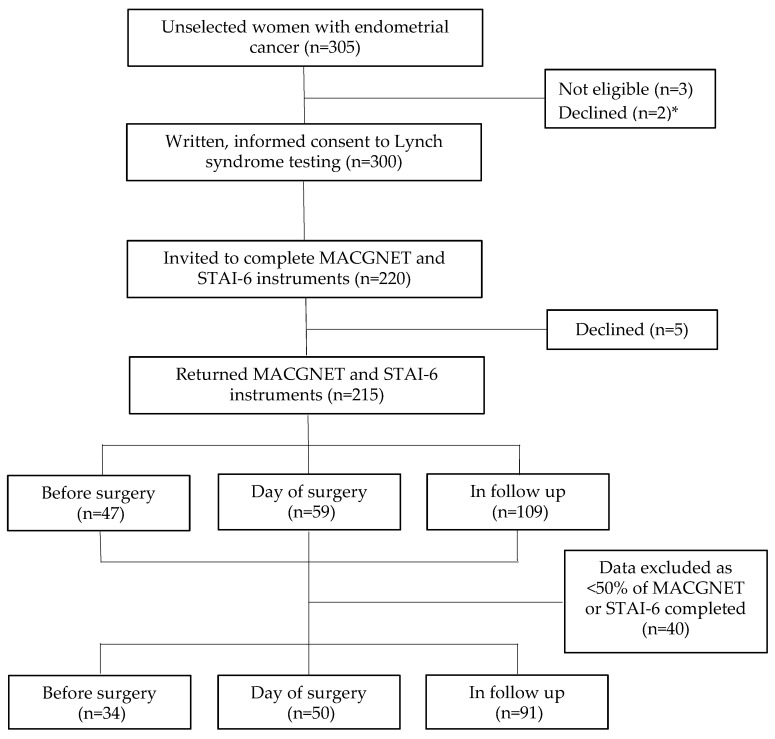
Study flow diagram. Footnote. * Three women initially declined Lynch syndrome testing however one later changed her mind.

In total, 305 women with endometrial cancer were offered Lynch syndrome testing (Figure 1). Three patients declined because they were anxious about their cancer diagnosis and impending surgery; all three had been invited to participate on the day of their hysterectomy. One woman changed her mind and enrolled in the study after surgery. One of the remaining two died shortly after surgery and the other was lost to follow up. Both were over 80 years of age and had no living first degree relatives.

The final study population comprised 300 women with a median age and BMI of 65 years (range 25, 88), and 31 kg/m^2^ (range 17, 70), respectively. Their ethnicity reflected that of the North West of England with 83% White, 11% Asian, 3% Black and 3% other. The final pathology showed atypical hyperplasia (2.3%), endometrioid (72%), serous (9.3%), clear cell (4.3%), carcinosarcoma (7%), mixed (3.7%) or dedifferentiated (1.7%) endometrial cancer. The majority were low grade (2.3% grade 0, 44.7% grade 1, 19.3% grade 2 and 33.7% grade 3) early stage tumours (2.3% stage 0, 74% stage 1, 12.3% stage 2, 10.7% stage 3, 0.7% stage 4). Eighty women did not receive a MACGNET or STAI-6 instrument to complete. Reasons included elapsed patient car parking time or transport pressures (*n* = 37), no available translator (*n* = 11) and clinic time pressures (*n* = 32). There was no significant difference in the baseline characteristics of those who were offered instruments and those who were not.

### 3.2. Time Taken to Consent for Lynch Syndrome Testing

The average time taken to consent for Lynch syndrome testing was 8 min (SD 5 min 20 s). There were five outliers (ROUT method [16] Q = 0.2%); on removing these, the average time taken was 7 min 36 s (SD 5 min 16 s). The average time taken to consent (once outliers were removed) for Lynch syndrome testing when this was done before surgery, on the day of surgery or during follow up was 6 min 29 s, 3 min 58 s and 10 min 18 s, respectively. Taking consent during follow up took significant longer than before or on the day of surgery (*p* ≤ 0.0001 and *p* ≤ 0.0001, respectively) because women asked more questions. Women over 60 years of age took less time to consent to testing than those under 60 years, with an average time of 6 min 54 s versus 8 min 13 s, respectively (*p* = 0.0003). Those with a positive family history did not differ than those without, taking 7 min 12 s and 7 min and 52 s to consent to testing, respectively (*p* = 0.3). Only one patient requested professional genetic counselling before agreeing to Lynch syndrome testing. Prior to this she had spent 48 min discussing such testing with the gynaecologist. Unfortunately, the duration of the discussion with the genetics counsellor was not recorded.

### 3.3. Baseline Characteristics of the Cohorts

Overall, 175 women completed the MACGNET and STAI-6 instruments (Figure 1). This included women consented before (*n* = 34), on the day of surgery (*n* = 50) and in follow up (*n* = 91). There was no significant difference in participant age, BMI, ethnicity or family history of cancer between these three groups (all *p* > 0.05). There were also no significant differences between the women who were and were not offered the questionnaire, nor between those who completed and failed to complete the questionnaire in age, BMI, ethnicity or family history of cancer (all *p* > 0.05).

### 3.4. Motivations for Testing

The reasons women gave for undergoing Lynch syndrome testing did not differ according to when they were asked, whether that be before, on the day of surgery or in follow up (Table 1). Most women were motivated to be tested in order to protect their family.

### 3.5. Anxiety about Testing

**Figure 2:**
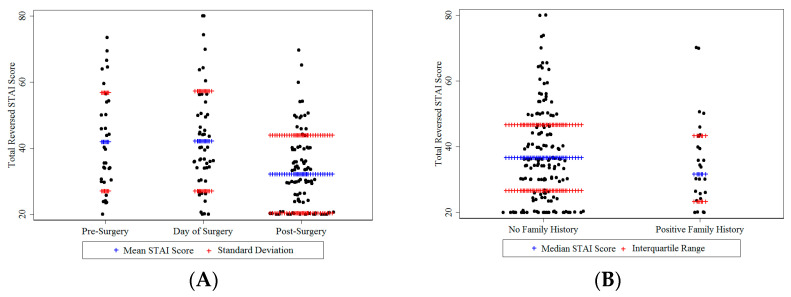
Reversed STAI-6 scores according to (**A**) when women were approached about Lynch syndrome testing and (**B**) their family history of cancer.

Anxiety levels were significantly lower when women were approached about Lynch syndrome testing in follow up [mean reversed STAI-6 score 32.1 (SD 11.8, range 20–70)] than before surgery [mean reversed STAI-6 score 42.0 (SD 14.9, range: 20–73.3)] or on the day of surgery [mean reversed STAI-6 score 42.2 (SD 15.1, range: 20–80)] (One-way ANOVA *p* < 0.001) (Figure 2).

Detailed family history was available for 173/175 participants. Two women were adopted and did not know their biological family. Of the 173 participants, 147 (85%) had no family history and 26 (15%) had a positive family history. The median reversed STAI-6 score for women with no family history was 36.7 (range 20–80) compared to 31.7 (range: 20–70) for those with a positive family history. The trend was for women without a family history to be more anxious about Lynch syndrome testing, although this was not statistically significant (Mann–Whitney *p* = 0.41) (Figure 2).

### 3.6. Lynch Syndrome Testing Outcomes

Thirteen of the 300 women in this study tested positive for Lynch syndrome. All were offered and received formal genetic counselling to discuss the implications of their diagnosis and opportunities to reduce their future cancer risk. Cascade Lynch syndrome testing was offered to at risk family members. At 12 months after the close of this study, 16 family members had tested positive, 16 negative and 3 had declined Lynch syndrome testing. There was no correlation between the timing of consent for Lynch syndrome testing and the success of cascade testing.

## 4. Discussion

Here we present the first study of gynaecologist led Lynch syndrome testing in women with endometrial cancer. The overwhelming majority of women (99%) accepted germline testing without the need for face-to-face professional genetic counselling. Women were motivated to undergo Lynch syndrome testing to protect their family, irrespective of whether they were approached before, at the time of surgery or in follow up after treatment for endometrial cancer. Anxiety scores were significantly lower when Lynch syndrome testing was sought during follow up but was not affected by family history status. Interestingly, the time taken to discuss testing and obtain written, informed consent was reduced if carried out in the pre-surgical setting.

The uptake of Lynch syndrome testing was much higher than previously reported [17,18,19]. This may reflect the NHS setting of our study wherein healthcare is publically funded and the personal implications for Lynch syndrome testing do not include raised insurance premiums. Only two women declined testing, principally because they were too anxious about their recent cancer diagnosis and impending hysterectomy. This is consistent with previous work examining uptake of genetic testing by parents of children with cancer [20]. Our findings are important because poor uptake of germline testing has led authors to call for caution regarding the implementation of universal Lynch syndrome testing in cancer populations [21,22]. Our study suggests that uptake is unlikely to be a significant barrier to gynaecologist led Lynch syndrome testing in the UK.

It took a little less than 8 min to discuss and obtain written, informed consent for Lynch syndrome testing in this study. This included time taken to explain the research proposal and for women to ask questions regarding participation in the study. The practicalities of incorporating consent into routine clinical care therefore needs further exploration, but our data suggest that allowing an extra 10 min or so in the setting of a routine gynaecology clinic should suffice. Whilst not trivial, since routine gynaecology clinics allocate 10–20 min/patient in the UK [23], it does mean that genetic counselling can be reserved for women who test Lynch syndrome positive. Thus the net resource implications are cost saving when compared to the traditional approach of genetic counsellors being the sole arbiters of germline testing [24]. There are implications for training; most gynaecologists are unfamiliar with discussing Lynch syndrome testing but good quality counselling is important to avoid misunderstandings, dissatisfaction and poor outcomes. We undertook formal training with genetic counsellors with expertise in the management of Lynch syndrome testing before consenting participants for this study.

Germline testing without prior genetic counselling has been advocated for other cancer predisposition syndromes [25], however, formal genetic counselling may reduce testing anxiety [26]. The average STAI-6 score for women approached during follow up in our study (32.1 SD 11.8) was lower than that reported by others when professional genetic counselling had been used. The work of Cull et al., Brain et al. and Watson et al. reported post genetic counselling STAI-6 scores of 33.7 (SD 9.8), 34.3 (SD 10.8) and 35.2 (SD 10.8), respectively [27,28,29]. It should be noted that our STAI-6 scores were significantly higher when women were approached shortly after diagnosis or at the time of hysterectomy. This infers that gynaecology led Lynch syndrome testing is feasible and does not cause unnecessary anxiety compared to the traditional model of prior referral for genetic counselling, so long as women are approached during follow up. Before surgery, they are already highly anxious and seemingly less receptive to discussions about hereditary risk and their future health. This finding is supported by previous work showing that highly anxious women were less likely to understand the information given to them about genetic risk; this in turn could be detrimental to their ability to give informed consent for testing [30].

Women were motivated to undergo Lynch syndrome testing to protect their family members and engage in prevention interventions. This was consistent whether consent was taken before surgery, on the day of surgery or during routine follow up. Although it did not reach significance, the MACGNET scores relating to prevention increased in women who were approached about Lynch syndrome testing during endometrial cancer follow up. This seems logical and adds further support to consenting women for Lynch syndrome testing after treatment, when they are better able to focus on future implications of having a cancer predisposition syndrome.

The strengths of our work include its novelty, the large number of women recruited and use of validated psychological instruments to identify motivators and stressors associated with gynaecologist led Lynch syndrome testing. Embedded in a non insurance-based healthcare system, our study provides an accurate measure of uptake unencumbered by any financial implications of being tested for a genetic condition. Limitations of our work include failure to offer questionnaires to all participants and incomplete responses from many. This may have introduced selection bias if responders were systematically different to non responders. Overall, 175 women provided complete responses and they did not differ from non responders in terms of key baseline characteristics; this provides reassurance that selection bias was minimal. The generalizability of our results to other Lynch syndrome-associated cancers, hereditary cancer predisposition syndromes and healthcare settings is unclear. Indeed, Balmaña et al. found significant differences in motivators for testing for different cancer predisposition syndromes, noting that individuals tested for Lynch syndrome were much more likely to be motivated by the potential to manage their future cancer risk than those tested for hereditary breast and ovarian syndrome (HBOS) [13,25].

## 5. Conclusions

Mainstream Lynch syndrome testing in endometrial cancer enables individualised treatment and provides opportunities to guard against future cancer by both the proband and her family members. Gynaecologist led testing is feasible and appears to be acceptable to women, especially when offered during routine follow up. Our work investigating cost effectiveness of gynaecologist led Lynch syndrome testing reveals cost savings that further strengthen the Manchester approach [24]. More research is now needed to confirm its effectiveness, including formal analysis of how well gynaecologists substitute for genetic counselors when consenting women for Lynch syndrome testing.

## Figures and Tables

**Table 1:** MACGNET scores exploring participants’ motivations for undergoing Lynch syndrome testing.

Questionnaire Subsection	Before Surgery (*n* = 34)	Day of Surgery (*n* = 50)	In Follow Up (*n* = 91)	*p* Value
Prevention Medical Care †	44 (42, 51), 35–55	46 (42, 52), 35–55	48 (44, 51), 18.7–55	0.19
Partners Influence †	12 (12, 16), 9–16	12 (12, 20), 9–20	13 (11, 16), 8–20	0.42
Future Planning	11.9 (1.9) 8–15	12.4 (2.0) 8–15	12.5 (2.5) 5–15	0.20
Ability to Cope	15.5 (3.2) 8–23	16.6 (4.1) 9–24	16.3 (4.3) 8–30	0.43

Results presented as mean score (standard deviation), minimum-maximum score, analysed by One-Way ANOVA. † Results presented as median (interquartile range), minimum-maximum score, analysed by Kruskal–Wallis statistical test.
